# Clinical outcomes for acute grade III and V acromioclavicular dislocations using hook plates and double-button fixation with a two-year follow-up

**DOI:** 10.1007/s00590-026-04842-7

**Published:** 2026-08-01

**Authors:** Erik Hohmann, Kevin Tetsworth

**Affiliations:** 1 Medical School, University of Pretoria, Pretoria, South Africa; 2 Royal Brisbane and Women′s Hospital, Brisbane, Australia

**Keywords:** Acromioclavicular joint, Acute dislocation, Clavicle hook plate, Double-button repair, Clinical outcomes.

## Abstract

**Purpose:**

To evaluate patient reported outcomes following combined ACJ stabilization using a clavicle hook plate and coracoclavicular augmentation with double-button fixation.

**Methods:**

This retrospective case series analyzed prospectively included all patients who underwent surgical treatment for isolated grade III or V acromioclavicular joint (ACJ) dislocation between 2021 and 2024. Inclusion criteria comprised age between 18 and 60 years and a minimum follow-up of 2 years. Associated traumatic injuries or fractures, and chronic cases defined in excess of more than three weeks from injury to surgery, were excluded. Clinical outcomes were assessed using the QuickDASH and Simple Shoulder Test (SST) scores.

**Results:**

Twenty-three patients (mean age 48.3 ± 10.2 years; mean follow-up 27.7 ± 2.8 months; *n* = 17 Type V, *n* = 6 Type III) were included. The mean preoperative QuickDASH score of 56.6 improved to 1.2 at 24 months, with 87% achieving MCID and SCB by 6 months and 100% reaching all clinical thresholds by 12 months. The mean SST score improved from 33.0 to 100. All patients reached MCID, SCB, and PASS by 12 months. Hook plates were removed at a mean of 5.6 months; four required early removal at 3 months due to pain and limited function. Two patients had mild superficial wound infections.

**Conclusions:**

Combined suture button and hook plate fixation in acute acromioclavicular joint dislocations results in excellent patient-reported outcomes, with return to normal shoulder function by 12 months. At six months, all patients met thresholds for MCID and SCB on the Simple Shoulder Test, while 87% achieved both benchmarks on the QuickDASH, indicating a high rate of clinically meaningful recovery.

**Level of evidence:**

Level IV; Case series.

## Introduction

Acromioclavicular joint (ACJ) dislocations are prevalent injuries, most commonly seen in athletes engaged in contact sports and in road traffic collisions [1]. The incidence of ACJ-dislocations is approximately 2 per 10,000 person-years, with Rockwood type III being the most common subtype, accounting for approximately 56% of all cases [2].

Stabilization of the ACJ is primarily provided by the coracoclavicular ligaments (CCL), comprising the conoid and trapezoid ligaments [3]. Injury to the CCL typically results in superior and horizontal displacement of the distal clavicle relative to the acromion [4]. In Rockwood type III and VI both the AC ligament and CCLs are completely ruptured, and surgical treatment is generally recommended [5].

The most commonly employed contemporary techniques for ACJ stabilization include arthroscopically assisted fixation using double-button suture devices inserted through transclavicular-transcoracoid tunnels, and clavicle hook plate fixation [5, 6]. Current evidence does not clearly support the superiority of one surgical technique over the other. The combination of suture button fixation and hook plate stabilization may offer the advantages of both techniques, providing enhanced mechanical stability, reduced immobilization duration, and facilitating early initiation of range of motion exercises and return to functional activities [7]. A recent meta-analysis demonstrated that while the addition of CC augmentation did not significantly improve functional outcomes or pain scores, it was associated with improved maintenance of reduction following implant removal and a 73% lower risk of acromial osteolysis [6].

The purpose of this study was to evaluate patient reported outcomes following combined ACJ stabilization using a clavicle hook plate and coracoclavicular augmentation with double-button fixation.

## Materials and methods

### Patient identification and data collection

**Fig. 1:**
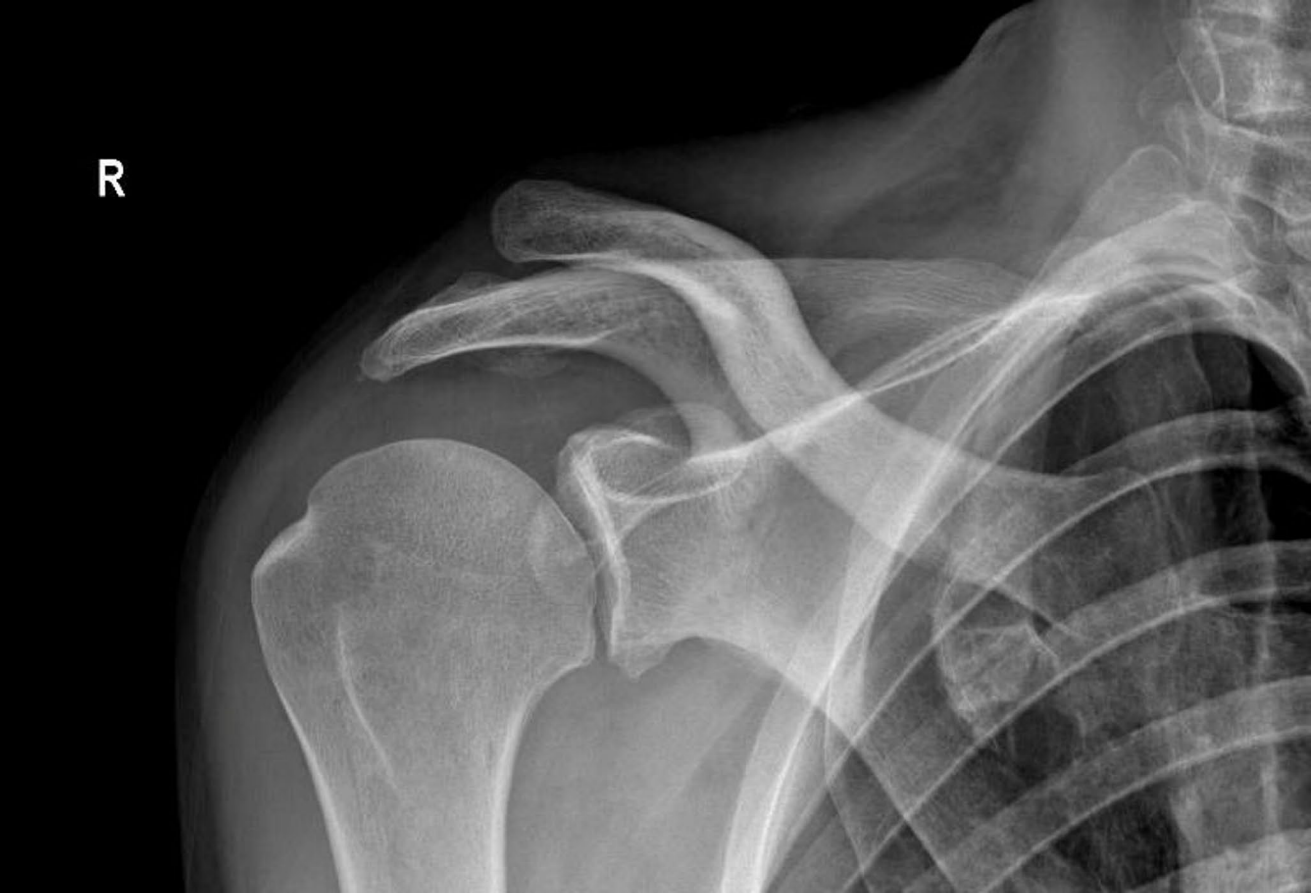
Rockwood Type V ACJ dislocation in a 57-year-old commercial pilot following a bicycle fall

This is a retrospective case series using prospectively collected data. The database of a single orthopedic surgeon was reviewed to identify patients who underwent surgical treatment between 2021 and 2024. Inclusion criteria were: isolated grade III or V ACJ dislocation (Fig. 1), age 18–60 years, minimum 2-year follow-up, and complete QuickDASH and Simple Shoulder Test (SST) scores. Patients with Rockwood type III injuries were included if they were high-demand individuals, including overhead athletes, manual labourers, or aviation personnel, in whom early restoration of full function and strength within a defined timeframe was required. We acknowledge that this selection criteria may introduce an element of selection bias. Rockwood Type III injuries were defined by superior displacement of the lateral clavicle, with the inferior cortex of the clavicle remaining at the level of, or just inferior to, the superior border of the acromion. In contrast, Rockwood Type V injuries were defined by marked superior displacement, in which the inferior cortex of the lateral clavicle lies well above the superior border of the acromion, reflecting complete loss of vertical stability. This method was selected over absolute coracoclavicular distance measurements to minimise measurement error related to radiographic magnification, patient positioning, and interobserver variability. Injury classification and assessment of reduction were based on restoration or disruption of inferior cortical alignment between the lateral clavicle and acromion, in keeping with the descriptive principles of the Rockwood classification system.

Exclusion criteria included additional trauma or fractures and chronic injuries with > 3-week delay from injury to surgery. This retrospective study was performed in accordance with the Declaration of Helsinki and institutional regulatory guidelines for human subjects research. Because this Level IV investigation analyzed exclusively de-identified, secondary clinical data from historical medical records, it was deemed exempt from formal review, and individual informed consent was not required. Patient confidentiality was strictly preserved using secure data encryption, and the study design introduced zero risk to past or present clinical care.

## Surgical technique

Patients are positioned in the beach-chair position, and anatomical landmarks are marked. After diagnostic arthroscopy via the posterior portal, an anteroinferior portal is established slightly medial and just above the subscapularis tendon. The coracoid undersurface is identified and cleared of soft tissue for visualization and preparation. The arthroscope remains for continuous intra-articular viewing. A 5 cm incision is made over the ACJ, extending medially along the clavicle. Sharp dissection proceeds to the clavicle’s superior surface. The coracoid is palpated, and anterior clavicular soft tissues are incised sharply. Dissection continues with blunt and sharp techniques to expose the coracoid’s superior surface.

A guide pin is inserted into the coracoid center, as close to its base as feasible, and advanced through it. The inferior exit is assessed arthroscopically and adjusted until central. It should be noted that precise placement at the base is not critical. As Molepo et al. showed, anterior positioning does not significantly affect horizontal displacement or construct stability [8]. Conventional jigs may produce eccentrically placed tunnels, acting as stress risers and increasing fracture risk [9]. To minimize cortical breach and ensure accuracy, a superior-to-inferior drilling technique is used, combining a superiorly centered guide pin under direct open visualization with arthroscopic confirmation of a centrally positioned inferior exit [9].

**Fig. 2:**
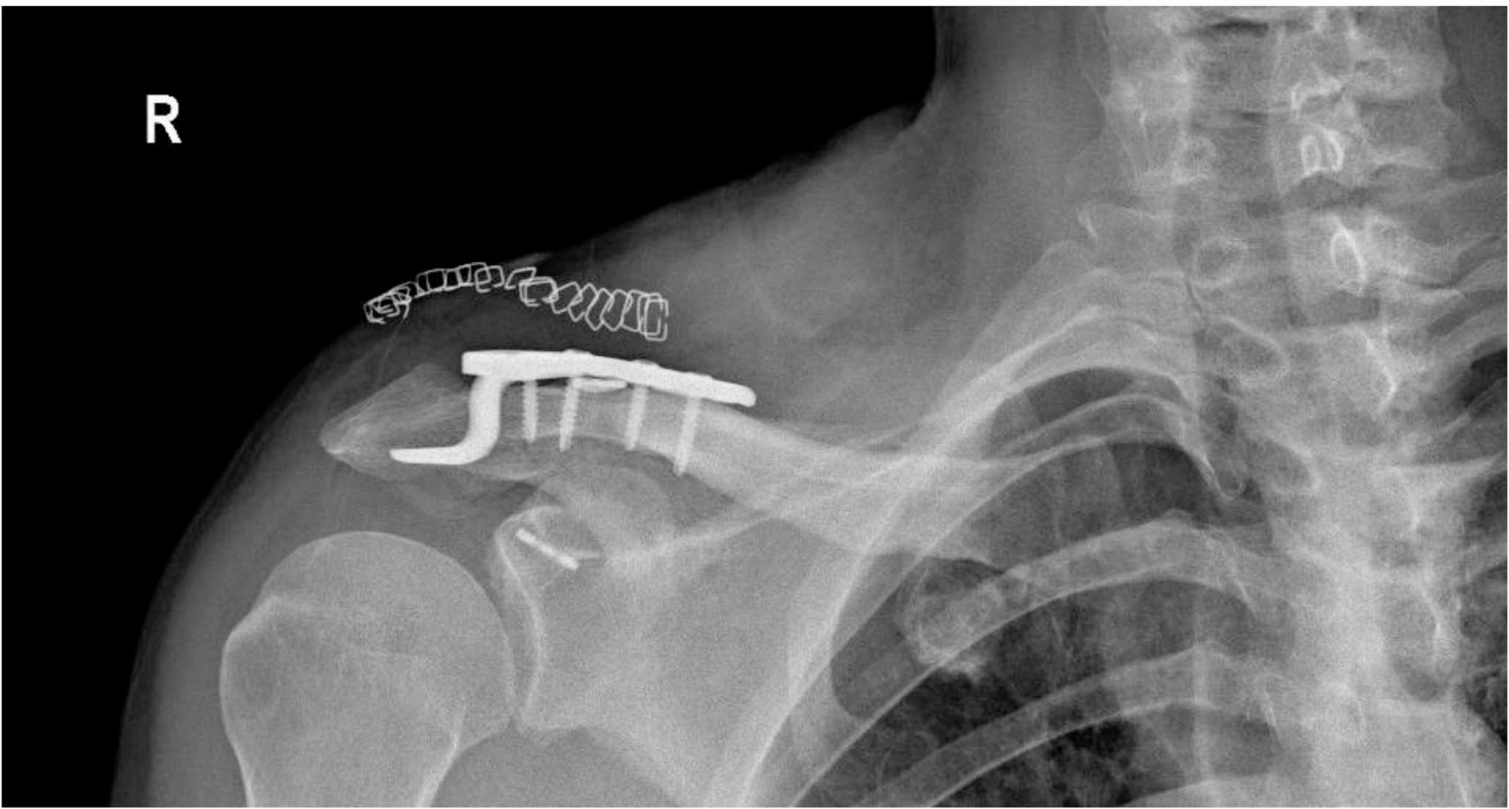
Postoperative radiograph of the same patient demonstrating fixation using a TightRope device in combination with a hook plate. Plate removal was typically scheduled approximately six months postoperatively but was performed earlier in cases where patients reported significant plate-related discomfort or irritation

A double-button device (Arthrex TightRope^®^) was shuttled through the clavicular and coracoid tunnels. The ACJ was anatomically reduced, and the TightRope construct tensioned and secured with multiple half-hitch knots for stable fixation. A suitably sized LCP clavicle hook plate was then positioned over the distal clavicle and fixed with 3.5 mm cortical screws (Fig. 2).

## Rehabilitation

Patients were initially immobilized in a shoulder sling for the first two weeks. Pendulum exercises and active-assisted range of motion exercises were encouraged during this period to promote early mobility and reduce stiffness. If tolerated, patients were permitted to discontinue sling use earlier and encouraged to move the arm freely within the limits of pain. A standardized physical therapy protocol was initiated two weeks postoperatively, with an initial focus on restoring range of motion. Progressive strengthening exercises were introduced at four weeks. Return to full activities, including manual work and sports, was typically permitted at three months postoperatively, based on functional recovery and clinical assessment.

## Outcome measures

The subjective outcome measures were the QuickDASH score [10] and the English version of the Simple Shoulder Test (SST) [11]. The QuickDASH shows strong psychometric validity and excellent test–retest reliability (Cronbach’s alpha > 0.94) [10], with intraclass correlation coefficients of 0.96–0.97 versus the full DASH [10]. The SST is reliable, responsive, and valid, with test–retest reliability > 0.9, minimal floor and ceiling effects, strong construct validity, and robust responsiveness [11].

Patient-reported outcome scores were collected by an independent research associate during follow-up visits or remotely via telephone or WhatsApp. The associate administered the questionnaires without influencing their completion. This approach minimized recording bias and the pleasing effect. Both questionnaires were completed at 6 weeks, and at 3, 6, 12, and 24 months after surgery.

Minimally important difference (MCID), patient acceptable symptom state (PASS), and substantial clinical benefit (SCB) were recorded for both scores. For the QuickDASH, MCID is 14 points [12], PASS is < 16 points [13], and SCB is an improvement ≥ 20 points [14]. For the SST, MCID is 2 points [12], PASS is > 10/12 [14], and SCB is an improvement > 3 points [15].

Radiographic outcomes were assessed on standard anteroposterior (AP) views at three predefined time points: immediately post-fixation, following plate removal, and at two years after the index procedure. The final radiograph at two years was used to assess reduction. Reduction was evaluated using the following criteria: alignment of the inferior cortex of the clavicle with the inferior cortex of the acromion along a continuous line, or residual superior displacement of the lateral clavicle of less than 5 mm.

### Statistical analysis

Descriptive statistics summarized demographic characteristics and PROMs. Mean age and standard deviation described demographics. For PROMs, 95% confidence intervals were calculated, and proportions achieving MCID, PASS, and SCB were reported as percentages. All analyses were performed using STATA SE 13.0 (StataCorp, College Station, TX, USA) and Comprehensive Meta-Analysis (CMA) version 3 (Biostat Inc, Englewood, NJ, USA).

## Results

Between January 2023 and January 2024, 23 patients underwent surgery using this technique, including six with Rockwood type III and 17 with type V ACJ dislocations. The mean age was 48.3 ± 10.2 years (range, 28–63), with 21 males and 2 females. Mean follow-up was 27.7 ± 2.8 months (range, 24–31). Demographics are summarized in Table 1.

**Table 1:** Patient demographics, mechanism of injury, and rockwood classification

Patient Nr	Sex	Age	Rockwood Type	Mechanism of Injury
1	Male	43	V	MBA
2	Male	58	III	MBA
3	Male	56	V	Cycling
4	Male	36	V	Fall
5	Male	49	V	Cycling
6	Male	58	V	Cycling
7	Male	40	V	MBA
8	Female	37	III	Rugby
9	Male	34	III	Rugby
10	Male	62	V	Fall
11	Male	57	V	Fall
12	Male	58	V	Cycling
13	Female	32	V	Cycling
14	Male	28	III	Rugby
15	Male	57	III	Cycling
16	Male	40	V	Fall
17	Male	52	V	Fall
18	Male	48	V	Cycling
19	Male	47	V	Cycling
20	Male	45	V	Hockey
21	Male	63	V	Cycling
22	Male	49	V	MVA
23	Male	61	V	Cycling

*MBA* motorbike accident, *Cycling* fall while cycling, *MVA* motor vehicle accident

## Clinical outcomes

### QuickDash

The preoperative mean score was 56.6 ± 12.6, improving to 1.2 ± 1.9 at 24 months. MCID, PASS, and SCB thresholds were progressively achieved, reaching 100% by 12 months (Table 2).

**Table 2:** Clinical outcomes during 24-month follow-up and plate removal timeline

Patient Nr	QuickDASH					SST					Plate removal
	6/52	3/12	6/12	12/12	24/12	6/52	3/12	6/12	12/12	24/12	
1	57	25	23	4	0	0	83	100	100	100	6/12
2	75	29	16	7	0	0	67	75	100	100	6/12
3	61	27	25	9	2	25	83	92	100	100	6/12
4	59	43	23	7	0	17	67	83	100	100	6/12
5	75	39	27	9	2	17	67	83	100	100	6/12
6	57	36	29	9	2	25	67	83	100	100	6/12
7	57	39	25	9	2	25	58	83	100	100	6/12
8	41	25	18	0	0	8	83	100	100	100	6/12
9	39	27	27	0	0	25	83	100	100	100	5/12
10	70	39	16	0	0	0	83	92	100	100	8/12
11	70	50	25	14	2	33	58	83	100	100	9/12
12	64	27	27	11	2	17	67	83	100	100	6/12
13	54	29	23	9	0	17	75	100	100	100	6/12
14	41	23	16	4	0	17	75	83	100	100	5/12
15	23	16	16	0	0	0	67	100	100	100	5/12
16	59	43	36	14	4	17	67	100	100	100	6/12
17	66	66	18	11	4	0	25	83	100	100	3/12
18	41	25	16	4	0	8	75	83	100	100	6/12
19	66	73	14	11	4	33	16	75	100	100	3/12
20	57	50	18	9	0	33	58	92	100	100	5/12
21	54	66	16	9	4	0	33	83	100	100	3/12
22	61	29	18	4	0	33	75	83	100	100	5/12
23	54	73	16	9	0	33	25	83	100	100	6/12
**Mean/SD**	**56.6 + 12.6**	**39.1 + 17.3**	**21.1 + 5.3**	**7.1 + 5.2**	**1.2 + 1.9**	**33 + 16.7**	**65.2 + 15.9**	**87.9 + 8.7**	**100**	**100**	5.6 + 1.6
**95% CI**	**51.1–62.0**	**29.0–44.0**	**18.0-22.6**	**4.6–9.1**	**0.4–2.1**	**11.2–22.0**	**59.7–73.4**	**83.4–90.9**	**100**	**100**	3.0–9.0
**MCID**		**78%**	**87%**	**100%**	**100%**		**83%**	**100%**	**100%**	**100%**	
**PASS**		**0%**	**0%**	**100%**	**100%**		**0%**	**39%**	**100%**	**100%**	
**SCB**		**39%**	**87%**	**100%**	**100%**		**83%**	**100%**	**100%**	**100%**	

### Simple shoulder test (SST)

The preoperative mean score was 33.0 ± 16.7, improving to 100% at 12 and 24 months, with MCID, PASS, and SCB achieved in all patients by 12 months (Table 2).

### Radiographic outcomes

Anatomic reduction was achieved in all patients and maintained after plate removal, with no displacement or TightRope migration at final follow-up.

### Plate removal and complications

The hook plate was removed at a mean of 5.6 ± 1.6 months. Four plates were removed at 3 months due to pain. Two patients had mild superficial wound dehiscence, resolving with antibiotics. Routine arthroscopy performed during hardware removal identified mild bone loss measuring less than 5 mm at the hook site in three patients who underwent early plate removal, accompanied by subacromial bursitis. These patients received an intraoperative subacromial corticosteroid injection, resulting in complete symptom resolution within three weeks. All patients reported significant subjective improvement in symptoms and function after plate removal; however, these benefits were not reflected in the clinical outcome scores.

## Discussion

The most important finding of this study is that combined ACJ stabilization using a clavicle hook plate and CC augmentation with a double-button device results in excellent patient-reported outcomes in ACJ dislocations. At six months postoperatively, the QuickDASH scores indicated that patients continued to experience mild residual disability, although 87% achieved both the MCID and SCB. In contrast, the SST demonstrated near-normal shoulder function at this time point, with 100% of patients meeting the thresholds for both MCID and SCB. By 12 and 24 months, all patients achieved scores consistent with full recovery, indicating a return to normal shoulder function on both outcome measures. These results demonstrate that the improvements are clinically meaningful and represent substantial patient-perceived benefits associated with the dual-construct stabilization technique.

Lee et al. conducted a meta-analysis that included one randomized controlled trial and four case-controlled studies to evaluate whether the addition of CC augmentation to hook plate fixation provides any clinical advantages in the treatment of acute acromioclavicular joint dislocations [6]. While no significant differences were observed in functional outcome measures, including the Constant, UCLA, and ASES scores, the authors concluded that CC augmentation offers mechanical benefits, specifically maintenance of reduction and a 73% reduction in the risk of acromial osteolysis [6]. Xie et al. conducted a systematic review including two randomized controlled trials and fourteen non-randomized studies comparing hook plate fixation with suture button constructs [5]. While some individual studies demonstrated potential advantages of suture button fixation such as higher Constant scores and lower VAS pain scores, the majority of included studies reported no statistically significant differences in functional outcomes between the two techniques [5].

In the present study, subacromial impingement and radiographic signs of osteolysis were observed in 13% of patients, all of which resolved following plate removal and was lower when compared to other studies. Huang et al. reported a pooled osteolysis rate of 29% in a meta-analysis of 32 studies evaluating hook plate fixation for acromioclavicular joint dislocations [16]. Akar et al. reported acromial erosion in 49% of patients undergoing surgical treatment for acromioclavicular joint dislocations [17]. Notably, the highest incidence of osteolysis was observed in patients with a type I acromion (26%), followed by those with type II acromion (16%), whereas patients with type III acromion demonstrated the lowest erosion rates at 7% [17].

Rehabilitation protocols following ACJ reconstruction with suture button fixation vary considerably across institutions and surgical techniques [18]. However, general recommendations include immobilization in a shoulder sling for approximately six weeks, with passive ROM exercises initiated around two weeks postoperatively and progression to active ROM by six weeks and strengthening exercises [21]. In contrast patients treated with a clavicle hook plate may begin mobilization earlier [19]. The shoulder sling can be discontinued as early as two weeks postoperatively, with the initiation of active range of motion and isometric strengthening exercises shortly thereafter [19]. This early mobilization is facilitated by the inherent mechanical stability provided by the hook plate construct [19].

A recent current concepts review identified over 400 publications addressing the management of acromioclavicular joint injuries [20], with a PubMed search revealing that 269 of these were published within the past decade. Numerous surgical techniques have been described, reflecting the evolving nature of treatment strategies [20]. Among the most widely adopted contemporary approaches are hook plate fixation and suture button constructs [21]. In the acute setting, suture button fixation has emerged as the preferred technique in many recent studies [21]. Current evidence indicates that open and arthroscopic acromioclavicular joint reconstructions using suture button fixation yield comparable outcomes in terms of reduction loss, complication rates, and revision surgery [22]. These findings highlight the ongoing debate and lack of consensus regarding the optimal surgical treatment for acromioclavicular joint dislocations. Current evidence suggests that both suture button fixation and hook plate techniques provide comparable functional outcomes and pain relief; however, suture button constructs may offer advantages in terms of lower hardware-related complications, such as reduced rates of osteolysis and fewer reoperations. Hook plates remain a viable option, particularly in cases where immediate mechanical stability is essential.

The combined use of suture button fixation and hook plate stabilization in this study appears to address the individual limitations of each technique. As demonstrated by Lee et al. [6], augmenting hook plate fixation with CC stabilization improved maintenance of reduction and significantly reduced the risk of osteolysis, although functional outcome scores did not show a clear advantage. The favorable results observed in the present cohort may be partly attributed to the early mobilization protocol, made possible by the enhanced biomechanical stability of this hybrid construct. The synergistic effect of dual fixation likely supports both the preservation of reduction and a more accelerated return to function [7].

The surgical technique employed in this study differs from conventional arthroscopically assisted reconstructions by utilizing a mini-open approach that allows direct visualization of the coracoid process, combined with intra-articular arthroscopic guidance. This hybrid method aims to enhance the accuracy of tunnel placement and mitigate the risk of coracoid fractures, a known complication linked to the use of traditional drill guide systems [8, 9]. One of the primary disadvantages of this hybrid approach is the need for routine hook plate removal, necessitating a second surgical procedure. The large number of surgical techniques described in the literature reflects the ongoing lack of consensus regarding optimal acromioclavicular joint reconstruction [20, 21, 23]. Accordingly, the present technique should be viewed as one of several viable options rather than a definitive solution.

In this study, the authors aimed to remove the hook plates at a minimum of six months postoperatively. Notably, all patients reported significant subjective improvement in pain and function following plate removal, despite these changes not being reflected in objective clinical outcome scores. This discrepancy highlights a potential limitation of PROMs, which may not fully capture subtle improvements in patient comfort and satisfaction. This also likely reflects the inherent differences between transient, event-related symptom changes and global outcome measures. PROMs are designed to capture overall pain and functional status over a defined recall period and may be less sensitive to short-term, focal improvements following implant removal. In this context, the observed subjective benefit after plate removal likely represents a local reduction in implant-related irritation rather than a measurable change in overall shoulder function, which may not be fully reflected in composite outcome scores.

Unlike the well-documented complications associated with suture-button fixation such as loss of reduction, implant migration, and coracoid fracture, no adverse events directly attributable to the double-button component were observed in this cohort. This may be due to the additional mechanical support provided by the concomitant hook plate, which likely contributed to the overall construct stability.

This study has several limitations. This study is a retrospective case series without a control group, which limits the ability to establish causal relationships or directly compare the combined technique to isolated hook plate or suture button fixation. The relatively small sample size, with a predominance of high-grade (Rockwood type V) injuries, may reduce the generalizability of the findings to broader patient populations. Although PROMs were systematically and comprehensively collected, the study did not include objective assessments such as radiographic evaluation of reduction maintenance, range of motion, muscle strength, or biomechanical stability.

It could be argued that the use of PROMs is insufficient and does not capture other variables such as range of motion and additional objective outcome measures. However, Hamilton et al. clearly emphasise that PROMs are not merely subjective opinions, but structured and standardised instruments that objectively quantify a patient’s pain, functional status, and disease severity from the patient’s perspective [24]. When appropriately developed and validated, PROMs provide robust and reproducible measurements and should therefore be regarded as objective outcome tools within clinical research. Furthermore, several studies have demonstrated correlations between shoulder range of motion, other clinical assessment tools, and PROMs [25]. PROMs should not be regarded as purely subjective feedback; rather, they are structured, psychometrically validated, and reproducible instruments for quantifying health status, often demonstrating reliability comparable to or exceeding that of manual clinical measurements [24]. As clinical success is ultimately defined by the patient’s perceived pain relief and functional capacity in daily life rather than isolated goniometric values or radiographic parameters, PROMs provide a robust and clinically meaningful assessment of surgical outcomes [24]. The inclusion of a selected subgroup of patients with Rockwood type III injuries, specifically those with high functional demands, including overhead athletes, manual labourers, and aviation personnel requiring early restoration of strength and function potentially introduced an element of selection bias and may limit the generalisability of the findings to the broader population of patients with type III ACJ injuries managed non-operatively or with lower functional demands. There was a disproportionate distribution of injury types, with Rockwood type V injuries accounting for 75% of the included patients. This imbalance may have introduced a degree of reporting bias and should be considered when interpreting the results. Another potential limitation of this study is the presence of a ceiling effect in the functional outcome scores. Given the generally high postoperative functional results, several patients achieved near-maximal scores, which may reduce the sensitivity of the scoring instruments to detect subtle differences at the highest levels of function. This limitation should be considered when interpreting the outcome data, as it may mask small but clinically relevant variations between patients. Furthermore, as this was a single-center study performed by a single surgeon with over 25 years of experience, the reproducibility of these results in different clinical settings or among less experienced surgeons remains uncertain.

## Conclusions

The results of this study suggest that combined suture button and hook plate fixation in patients with acute acromioclavicular joint dislocations results in excellent patient-reported outcomes, with a return to normal shoulder function achieved by 12 months. At six months postoperatively, all patients met the thresholds for the Minimal Clinically Important Difference and Substantial Clinical Benefit on the Simple Shoulder Test, while 87% achieved both benchmarks on the QuickDASH, indicating a high rate of clinically meaningful recovery.

## Data Availability

No datasets were generated or analysed during the current study.
